# The public health issue of antibiotic residues in food and feed: Causes, consequences, and potential solutions

**DOI:** 10.14202/vetworld.2022.662-671

**Published:** 2022-03-23

**Authors:** Mbarga Manga Joseph Arsène, Anyutoulou Kitio Linda Davares, Podoprigora Irina Viktorovna, Smolyakova Larissa Andreevna, Souadkia Sarra, Ibrahim Khelifi, Das Milana Sergueïevna

**Affiliations:** Department of Microbiology and Virology, Institute of Medicine, RUDN University, Moscow, Russia

**Keywords:** animal breeding, antibiotic resistance, antibiotics residues, food and feed, public health

## Abstract

Antibiotics are among the essential veterinary medicine compounds associated with animal feed and food animal production. The use of antibiotics for the treatment of bacterial infections is almost unavoidable, with less need to demonstrate their importance. Although banned as a growth factor for a few years, their use in animals can add residues in foodstuffs, presenting several environmental, technological, animal health, and consumer health risks. With regard to human health risks, antibiotic residues induce and accelerate antibiotic resistance development, promote the transfer of antibiotic-resistant bacteria to humans, cause allergies (penicillin), and induce other severe pathologies, such as cancers (sulfamethazine, oxytetracycline, and furazolidone), anaphylactic shock, nephropathy (gentamicin), bone marrow toxicity, mutagenic effects, and reproductive disorders (chloramphenicol). Antibiotic resistance, which has excessively increased over the years, is one of the adverse consequences of this phenomenon, constituting a severe public health issue, thus requiring the regulation of antibiotics in all areas, including animal breeding. This review discusses the common use of antibiotics in agriculture and antibiotic residues in food/feed. In-depth, we discussed the detection techniques of antibiotic residues, potential consequences on the environment and animal health, the technological transformation processes and impacts on consumer health, and recommendations to mitigate this situation.

## Introduction

Antibiotics are among the essential veterinary medicine compounds related to food animal production, defined as substances that can kill or inhibit the growth of bacteria [1]. Its application is almost unavoidable in the treatment of bacterial infections in animals and humans [2]. The global consumption of antibiotics in animals is almost twice that of humans [3]. Globally, 63.1±1.5 tons of antibiotics are annually used in livestock [4], estimating that over 80% of the animals for food production are currently being treated with these compounds [5].

Antibiotics help treat and prevent various infections in animals, such as mastitis, arthritis, respiratory diseases, and gastrointestinal and other bacterial infections [1], and are used as a growth factor with imminent consequences [6]. However, if they are misused, they can end up as residues in foodstuffs, such as milk, eggs, and meat, thus exerting harmful effects on the health of consumers [7]. Furthermore, among the different forms of administration (oral, parenteral, or topical), it was reported that antibiotic residues exceeding the standards are usually encountered when administered through injection [8]. Therefore, various authors have reported that antibiotic residues in food are likely to induce and accelerate the development of antibiotic resistance in bacteria, promote the transfer of antibiotic-resistant bacteria to humans, cause allergies (penicillin), and induce other more severe pathologies, such as cancers (sulfamethazine, oxytetracycline, and furazolidone), anaphylactic shock, nephropathy (gentamicin), bone marrow toxicity, mutagenic effects, and reproductive disorders (chloramphenicol) in humans [1].

Therefore, the World Health Organization (WHO), the American Medical Association, and the American Public Health Association have urged a ban on growth-promoting antibiotics [9] and established standards to limit this phenomenon [10]. Consequently, drugs or antibiotic residues in food above the maximum level globally recognized by various public authorities are illegal [10,11]. Furthermore, observation of the waiting or withdrawal time and physicochemical analyses is mandatory to ensure that the antibiotics used or their analogs do not exceed the maximum residue limit (MRL) before the food is marketed.

With regard to the issue of residues as a topical public health concern and the excessive growth of antibiotic resistance over the years [12]. This review, discussed the regular application of antibiotics in agriculture, antibiotic residues in food and feed, detection techniques, potential consequences on the environment and animal health, technological transformation processes and impacts on consumer health, and recommendations to mitigate these situations.

## Review Methodology

This review article was conducted by exploiting numerous review articles, original articles, and related books from reputable databases, such as Web of Science, PubMed, and Scopus. The papers published with toll access have been made available using the facilities provided by the Peoples’ Friendship University of Russia, Moscow, Russia. The literature investigation process was conducted in English and French between October 2020 and April 2021. The keywords explored during the literature search consisted of combinations of the following words: “antibiotic,” “animals,’’ “residue,” “food,” “antimicrobial drugs,” “detection,” “meat,” “milk,” “eggs,” “antibiotique,” “animaux,” “résidus,” “aliments,” and “médicaments antimicrobiens.”

## The Common Application of Antibiotics in Agriculture

Antibiotics are used for food production for several reasons, including their primary use as preventive or curative measures against animal infection. Others are used as growth promoters for improved feed conversion efficiency, carcass quality, and economic production [4]. However, in the use of antibiotics in agriculture on a larger scale, the first property is its safety and effectiveness [1]. This property is essential because it aims to protect consumers from severe infections transferrable to humans through contact with the infected animal, consumption of contaminated food, or proliferation in the environment [13]. The frequently used antibiotics in veterinary medicine are b-lactams (penicillin and cephalosporin), macrocyclines (ansamycins, glycopeptides, and aminoglycosides), tetracyclines, chloramphenicols, macrolides, spectinomycin, lincosamide, sulfonamides, nitrofurans, nitroimidazoles, trimethoprim, polymyxins, and quinolones [14]. However, Bacanlı and Başaran [1] reported that the combination of streptomycin and oxytetracycline or streptomycin alone could sometimes be used during the treatment and prophylaxis of plant diseases. Although its main purpose is to fight against pathogens, antibiotics are used explicitly with the disease of concern to be treated. According to the breed, the bacteria responsible for infections vary from one animal to another. The choice of antibiotics and the antimicrobial consumption pattern demonstrate geographical variation across the continents influenced by the food animal species, regional production patterns and types of the production system, intensive or extensive farming, the purpose of farming (commercial or industrial or domestic), unclear legislative framework or policies on the use of antibiotics, and size and socioeconomic status of the population and farmers in particular [15].

## Antibiotic Residues

### Some published work showing the presence of antibiotic residues in common foods

Table-1 recorded some studies that reported antibiotic residues in food [16-47]. These foods are mainly obtained from poultry (chicken meat and eggs), bovines (bovine carcasses, milk, and meat), and pigs (pork meat). We also reported at least one positive case per continent. However, interestingly, during the literature investigation, we found it easier to obtain work reporting the presence of antibiotic residues in developing countries (mainly in African countries) than in developed countries. This trend is attributed to the lack of legislation and its non-strict application [48], non-systematic detection of residues [49], and improper use or use without a prescription of veterinary antibiotics [15-17].

**Table-1:** Antibiotic contamination in various foodstuffs consumed in different countries.

Country	Antibiotic	Foodstuff	Reference
America	Tetracyclines	Imported Chicken Meat	[18]
Brazilia	Tetracyclines, fluoroquinolone		
Bangladesh	Amoxicillin	Milk, eggs	[19]
Cameroon	Tetracyclines	Chicken	[16]
China	Quinolones, tetracyclines, sulfonamides	Chicken, chicken giblets, and eggs	[20]
	Beta-lactams, tetracyclines, sulfonamides, and quinolones	Milk	[21]
Egypt	Tetracyclines	Bovine carcasses	[22]
	Tetracyclines	Chicken meat	[23]
	β-lactams Cephalosporines	Eggs	[17]
	β-lactams Cephalosporines	Rabbit meat Rabbit liver Rabbit kidney	[24]
Ethiopia	Tetracyclines	Meat and edible tissue	[25]
Ghana	Tetracyclines	Milk	[26]
Greece	Nitrofuran	Pork	[27]
India	Oxytetracycline and erythromycin	Honeys	[28]
	Enrofloxacin, oxytetracycline penicillin G and sulfamethoxazole	Milk	[29]
India	Tetracycline, Oxytetracycline, Sulfadimidine, Sulfamethoxazole	Raw milk	[30]
Iran	Penicillin, chloramphenicol, gentamicin, tylosin, tetracycline, and sulfonamide	Honey	[31]
Italy	Nitrofuran	pork	[27]
Kenya	Tetracyclines	Beef, liver and kidney	[32]
	Tetracyclines, sulfamethazine, beta-lactams, and gentamicin	Milk	[33]
	b-lactams	Milk	[34]
Malaysia	Sulfonamides	Chicken	[35]
Mexico	Penicillin	Milk	[36]
Nigeria	Tetracyclines	Meat	[37]
	Tetracyclines	Eggs	[38]
	Clhoramphenicol	Eggs	[39]
	b-lactams	Cattle meats	[40]
Portugal	Nitrofuran	Pork	[27]
South Africa	Ciprofloxacin, streptomycin, tetracycline, and sulfanilamide	Beef; chicken; pork	[41]
	Tetracycline	Chicken livers	[42]
Sudan	Macrolides	Milk	[43]
Tanzania	Tetracyclines	Milk	[44]
Tanzania	Tetracyclines	Eggs	[45]
Turkey	Quinolones	Chicken, beef	[46]
Zambia	Oxytetracycline and Sulfamethazine	Beef	[47]

## Movements of Antibiotic Residues through the Animal–human Interface

**Figure-1:**
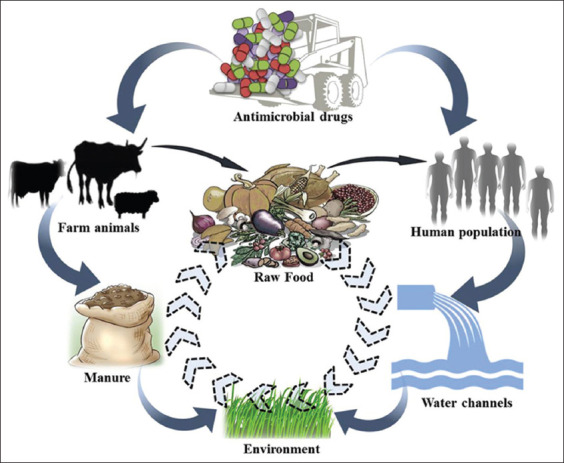
Conceptual representation of possible movement of antibiotic residues between different ecosystems [50].

Several factors occur before antibiotic residues are found in food. We adapted the conceptual representation proposed by Sharma *et al*. [50] (movement of antibiotic-resistant bacterial strains/genes between different ecosystems) because we judged that the movement of residues obeyed a similar dynamic. Figure-1 [50] shows that any direct or indirect interaction between humans and animals may cause antibiotic residue transmission. Direct interaction occurs when the antibiotics given to the animals or applied to plant cultures become directly residual in the foodstuffs after slaughter or harvest. On the contrary, direct interaction occurs when the residues accumulated in water and soil through manure (containing antibiotic residues from animals) or human excretions (containing residues of antibiotics) are found in food (especially in vegetables by watering or contamination with animal feces) [51]. Although the latter is unlikely to occur compared with the former, several studies have reported significant amounts of antibiotic residues in manure in soils, concluding that these residues can end up in plant foods [52-56]. For the direct mechanism, it is important to stress that the types and amounts of antibiotics administered to the animals also play a significant role, including the mode of administration. Apparently, among the different forms of administration (oral, parenteral, or topical), it has been reported that antibiotic residues exceeding the standards are mainly encountered when administered through injection [8]. This mode of administration might cause the accumulation of drugs in the adipose tissue, limiting the metabolism and the elimination of this drug, which could therefore make them persist in the tissues of animals even after slaughter. 

## The Main Techniques for Detecting Antibiotic Residues

The methods for detecting antibiotic residues can be divided into two categories: screening method (1) and confirmatory method (2). These two methods differ because the first one is globally qualitative or semiquantitative. In contrast, the second one ensures its determination with high precision of the type and concentration of the investigated residue.

## Screening Method

All screening methods are essentially microbiological or immunological. The most well-known and earliest microbiological technique is the so-called “four plates” technique. This method inhibits the growth of *Micrococcus luteus* and *Bacillus subtilis*. Inhibitory zones lacking bacterial colonies around the sample deposit sites indicate the potential presence of antibiotics [57]. This method is widely used to investigate the presence of antibiotic residues in different meats, fish, and eggs [57-60]. Similarly, acidification is also a well-known microbiological technique used to detect the presence of antibiotic residues in milk. This test uses a culture of a bacterium capable of degrading lactose into lactic acid and a colored indicator, bromocresol purple, which detects if the acidification of the medium has been done. The most suitable strain for this method is *Bacillus stearothermophilus* var. *calidolactis* C953 (strain C953, CIP 5281) [57].

Furthermore, if the milk analyzed contains antibiotics, the bacteria will not degrade the lactose, and the color of the medium will remain unchanged. On the other hand, the absence of antibiotics results in a color change from blue to yellow, indicating acidification. However, despite these techniques being accessible, inexpensive, and performed by a nonprofessional, there is a lack of specificity and long incubation time. Thus, to overcome these disadvantages, several companies have manufactured different commercial kits under different trade names (e.g., BR test, Eclipse test, Copan test, Delvotest, Lumac, and Arla). Such tests detect numerous antibiotics at thresholds generally close to the MRL [14].

Compared with microbiological methods, immunological methods such as enzyme-linked immunosorbent assay, fluoroimmunoassay, and time-resolved fluoroimmunoassay are highly specific, highly sensitive, simple, and cost effective. In addition, rapid-detection immunochromatographic kits (on strips) have been developed (TwinSensor), allowing within one assay the simultaneous screening of penicillins and cephalosporins (TwinSensor KIT034); beta-lactam and tetracycline (TwinSensor KIT020); beta-lactam antibiotics tetracyclines, streptomycin, and chloramphenicol (4Sensor BSCT-KIT060); beta-lactam antibiotics, sulfonamides, tetracyclines, and (Fluoro) quinolones (4Sensor BSTQ-KIT072) [61-64].

## Confirmatory Methods

The main advantage of confirmatory methods is their high specificity, but they are expensive, time-consuming, and require personnel and an adequate laboratory [1]. Confirmatory methods are essentially chromatographic methods (mainly liquid chromatography) coupled to mass spectrometry or ultraviolet (UV) [1]. However, capillary electrophoresis (CE) [65], CE–laser-induced fluorescence [66], surface-enhanced Raman spectroscopy [67], and high-performance liquid chromatography (HPLC) with spectroscopic fluorometric detection (HPLC– RF) or with a spectroscopic HPLC–photodiode array detector [42] are also shown to be effective detectors of antibiotic residues.

## New Fully Automatic Approach

Fully automatic biosensors are becoming increasingly important in detecting antibiotics in food [1]. The biosensors can be classified according to the biological element (enzymatic, immunosensory, and microbiological), transducer (piezoelectric, electrochemical, optical, thermal, impedimetric, and calorimetric), and biological element immobilization procedure on solid support (adsorption, covalent bonding, cross-linking, entrapment, and encapsulation) [14]. Cháfer-Pericás *et al*. [14], in their work, reported most of the existing techniques for the rapid detection of antibiotic residues in foods. They concluded that fully automatic biosensors consist of a combination of biological element/transducer(such as microbiological cell/electrochemical, antibody/impedimetric, and oligonucleotide/electrochemical with microbiological cell) represent an interesting screening approach due to its quick and fully automated operability [14]. They also reported the advantages of biosensors, specifically the biorecognition element used, which allows rapid, continuous control, and onsite applications. However, the main limitations of these instruments are (1) the potential loss of stability of the biological detection component due to exposure to environmental stresses, such as pH, temperature, or ionic strength, and (2) the limited size of the physicochemical transducers used.

## National and International Legislation on Antibiotic Residues in Food

**Figure-2:**
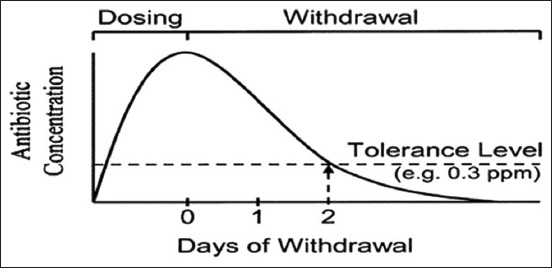
Theoretical representation of withdrawal period [68-70].

It is challenging to control the use of antibiotics in agriculture in a uniform way because their use varies significantly from one country to another . For example, it was reported that China is the first country to use antibiotics in food animals (23%), followed by the United States (13%), Brazil (9%), India (3%), and Germany (3%) [4]. This statistic seems disproportionate because it assumes that other countries use fewer antibiotics than the five countries. However, international and national regulatory agencies, such as Food and Agriculture Organization/WHO, Food and Drug Administration, Canadian Food Inspection Agency, the Australian Pesticides and Veterinary Medicines Authority, European Commission, European Food Safety Authority, and the Ministry of Health of each country, continuously attempt to regulate antibiotic use with international standards, considering the specific realities of each country. This harmonization mainly involves the control of parameters, such as (1) acceptable daily intake (ADI), which is a critical standard set from toxicological studies based on the no-observable-effect level and safety factor [68]; (2) withdrawal period or waiting time (WT), which refers to the minimum period from the administration of the last dose of medication and the production of meat or other animal-derived products for food (Figure-2) [68,69]; and (3) MRL, which is the highest level of an antibiotic residue or its metabolites that is legally tolerated in food when antibiotics are correctly applied following Good Agricultural Practice [20]. Although the ADI, WT, and MRL for most antibiotics have been established (for each food) and efforts have been made to regulate the MRL worldwide under the aegis of the World Trade Organization and the Codex Alimentarius, MRLs still vary from one geographical location to another. Meanwhile, although this situation seems to be under control in the European Union countries and other developed countries [68], the problem of antibiotic residues remains topical with unprecedented danger in developing countries due to the lack of control mechanisms despite the existing legislation.

## Consequences of the Presence of Antibiotic Residues in Food and Feed

**Figure-3:**
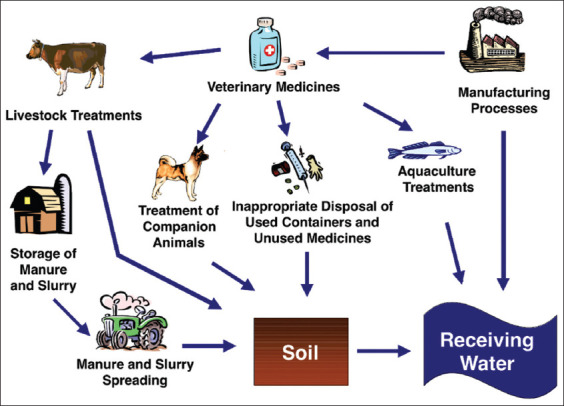
Potential pathways for veterinary medicines in soil and water [70].

Adverse consequences of antibiotic residues can be seen on four levels (Figures-2 and 3) [70]: (1) On the health of the animals themselves, (2) on the environment, (3) on the transformation processes (technological risks), and (4) on consumer health.

## Consequences and Risks on Animal Health

We will not discuss the consequences of antibiotics on animals, only those of their residues. It is well established that antibiotics administered to animals can be found in their feces [52-56]. The bacteria present in these feces or those responsible for infections are likely to be exposed to low doses of antibiotics, making them more virulent and more resistant [2,12,71,72] and leading to significant losses [73]. The animal digestive flora disturbance may also lead to the same result.

## Environmental Risks

The indiscriminate and improper use of antibiotics can result in higher concentrations of antibiotics in the environment, which is referred to as antibiotic pollution [15]. Most definitely, it is estimated that approximately 75% of antibiotics are not absorbed by animals but are excreted as waste [74]. In addition to this source, as presented in Figure-3, other factors in the production chain, including manure and slurry spreading, aquaculture treatment, and inappropriate disposal of used containers and unused medicines, can lead to the dumping of antibiotic residues in water and soils [70]. Recently, residues of antibiotics like fluoroquinolones (e.g., ciprofloxacin) and sulfonamides (e.g., sulfamethoxazole), which are chemically stable, are often detected in the environment, and the resistance to these antibiotics is frequently reported [75]. These residues are significantly considered pollutants since they disturb the normal flora of soils and water, consequently leading to the production of resistant bacteria through selective pressure. The mechanism of resistance acquisition is mainly attributed to the selection of resistance genes capable of causing enzymatic degradation of antibiotics by bacteria [76], modification of the target of the antibiotic [77], change in membrane permeability [78], and establishment of alternative metabolic pathways [79]. These resistance genes in bacteria can subsequently be transmitted to the next generation of bacteria through a vertical (from one generation to another) or horizontal (from one bacterium to another through a plasmid) gene transfer [75].

## Technological Risks

The main technological risks presented by antibiotic residues in foodstuffs are applied to products processed by microbial fermentation, such as milk (fermented milk products and cheese), meat (chorizo, corned beef, pepperoni, soudjouk, salami, and sausage), and fish (bagoong, fesikh, garum, gravlax, shrimp paste, fish sauce, and surströmming). Indeed, antibiotic residues in these raw materials can interfere with the fermentation process by inhibiting the starter cultures, leading to manufacturing accidents and poor-quality foods.

## Consequences on Consumer Health

Antibiotic residues are a severe public health issue [80], with their presence in foods capable of causing mild to adverse complications that are difficult to manage. Therefore, we have divided the toxic consequences into two subgroups: (1) Direct toxicity and allergic reactions and (2) resistance to antibiotics as indirect consequences.

## Direct Toxicity and Allergic Reactions

### Allergic reactions

Recently, several studies have reported that antibiotic residues can elicit allergic reactions. Most of the reported allergies are related to beta-lactam antibiotic residues, especially penicillin and cephalosporins. They include skin rashes, serum sickness, thrombocytopenia, erythema multiforme, hemolytic anemia, vasculitis, acute interstitial nephritis, Stevens–Johnson syndrome, and toxic epidermal necrolysis [81]. For example, allergic reactions have been reported in people who consumed milk [48,81], meat [82], and pork [83], all containing penicillin residues. Furthermore, some studies have mentioned that aminoglycoside, sulfonamide, and tetracycline residues can also cause allergic reactions [1,84].

### Hepatotoxicity

Limited data exist on the consequences of antibiotic residues on the liver, possibly due to a lack of investigations pointing toward this direction. However, the hepatotoxic effects of some antibiotics are well known. For example, Hautekeete [85] reported that penicillin, oxacillin, cloxacillin, flucloxacillin, and amoxicillin-clavulanate could cause hepatitis (mainly cholestatic). He also reported that tetracyclines could cause a syndrome mimicking acute fatty liver of pregnancy, indicating the potential of erythromycin and several other macrolides to cause hepatitis (usually cholestatic) [85]. Furthermore, Van Gerven *et al*. [86] and Hautekeete [85] reported that nitrofurantoin could cause chronic hepatitis mimicking chronic autoimmune hepatitis acute cholestatic and hepatocellular reactions. Finally, it has been reported that ceftriaxone can cause drug-induced gallstones and quinolone cholestasis, and sulfamethoxazole/trimethoprim can cause severe hepatotoxicity, especially in patients with acquired immunodeficiency syndrome [85]. Consequently, we can hypothesize that the residues of the aforementioned antibiotics, whose hepatotoxic effects are well known, can also have harmful consequences on the liver if present in high concentrations.

### Destruction of normal and/or useful intestinal flora and indigestion

The intestinal flora contains several microorganisms (with nearly 1000 species) that play an important role in human physiology and health [81]. Despite the inadequate information on the direct impact of residues of antibiotics in food, Kyuchukova [81] and Beyene [80] pointed out that a broad spectrum of antibiotics used in feed can end up in food, adversely affecting the intestinal flora and subsequently causing the gastrointestinal disturbance.

### Mutagenicity, reproductive disorders, and teratogenicity

In a recent study summarizing data from 73 scientific studies reporting antimicrobial residues in animal products readily available for sale, Treiber and Beranek-Knauer [87] highlighted that the frequency of mutations is increased with antibiotic residues. In addition, Botsoglou and Fletouris [88] found that several drugs, including doxorubicin, elicit mutagenic activities. Similarly, Beyene [80] reported antibiotic residues as a probable threat to the human population as they adversely affect human fertility. Finally, in their reviews of the consequences of antibiotic residues on humans, Kyuchukova [81] and Darwish *et al*. [48] mentioned that antibiotic residues could produce reproductive disorders and teratogenic effects.

### Carcinogenicity and other effects

Some antibiotic residues, such as sulfamethazine, oxytetracycline, and furazolidone, have carcinogenic effects [1,48,87]. Aside from their carcinogenicity, other effects, including bone marrow toxicity (mainly due to chloramphenicol) and nephropathy (mainly due to gentamicin), were also reported [1]. Beyond all these adverse effects, antibiotic resistance, an indirect consequence of antibiotic residues in food, is the most catastrophic issue, with the WHO estimating that if nothing is done to address this problem, drug-resistant diseases may cause 10 million deaths each year by 2050, consequently damaging the economy as catastrophic as the 2008-2009 global financial crisis [89].

## Resistance to Antibiotics

The current transmission mechanisms of antibiotic resistance are better understood than those in the past. It has been established that the exposure of bacteria to low doses of antibiotics is likely to lead to bacterial adaptation, making them more resistant and more virulent [90-94]. Since antibiotic residues can be considered low or subtherapeutic doses, we assume that bacterial exposure can lead to adaptation. This mode of resistance acquisition is mainly based on spontaneous mutations and positive selection [95,96], alteration of the target with decreased affinity for the antibiotic [77,96], alternative metabolic pathways [79], change in membrane permeability, and efflux pumps [78,91]. The transmission of resistance between bacteria through a vertical (from one generation to another) or horizontal (transfer of resistance genes from one bacterium to another through conjugative plasmids, which play an essential role in the dissemination of resistance genes) gene transfer has worsened the situation [75], given that any direct or indirect interaction between humans and animals may lead to zoonotic transmission of antibiotic-resistant strains and genes from food animals to humans (Figure-1) [50]. Therefore, antibiotics should be carefully used to reduce the risks of resistance development and their complications in the management of diseases induced in humans. The most resistant germs found in breeding and food, which are implicated the most in these phenomena, are essentially resistant salmonellae, glycopeptide, or streptogramin-resistant enterococci, multiresistant *Escherichia coli*, and macrolide or fluoroquinolone-resistant campylobacters [1]. Since carbapenems are strictly prohibited in food-producing animals, the carbapenemase gene plasmids are not expected to end up in the food chain. However, studies reported the presence of carbapenem resistance on plasmids in animal breeding, such as blaOXA-181 in pigs from Italy, blaNDM-1 in pigs, and blmNDM-17 in chicken from China [1]. This result validates the disproportion in the use of antibiotics in some countries. The studies conducted worldwide report an increasing and alarming number of resistance genes to antibiotics in livestock each year. Therefore, measures must be taken to curb this phenomenon because the consequences of infections in humans by multidrug-resistant bacteria are highly expensive and often result in death.

## Potential Solutions and Recommendations

Solutions to the problems of antibiotic residues in foods require the implication of both producers and legal organizations. With regard to the responsibilities of legal organizations, we suggest (1) that more rigorous control over the types and concentrations of antibiotics used should be established, (2) antibiotics should be marketed only by professionals who should sell them only with a prescription from a veterinarian, (3) prohibition of antibiotics whose toxicity is established (i.e., chloramphenicol, furazolidone, nitrofurazone, sulfonamides, and fluoroquinolones) and those more likely to induce direct or cross-resistance to antibiotics, (4) mandatory quality controls of food before marketing and the fight against black market products, and (5) permanent awareness of the dangers posed by antibiotic residues in food and resistance to antibiotics. Similarly, producers must (1) use antibiotics only when necessary and under the prescription of a veterinarian, (2) respect withdrawal times and other good practices related to antibiotics, (3) systematically test the presence of residues of the antibiotics used in their production, and (4) educate themselves on the regulations in force and respect them. In a similar manner, economic and straightforward field tests should be developed to identify antibiotic residues in animal products quickly. Meanwhile, methods such as heat treatment, activated charcoal, resin, and UV irradiation, may help inactivate antibiotics. Finally, probiotics and active phytochemical compounds should be further investigated and used as alternatives to antibiotics.

## Conclusion

The residues of antibiotics in food are of severe public health concern. Although necessary, even essential in agriculture, they should nevertheless be used more sparingly to avoid the consequences (direct toxicity and resistance to antibiotics) they can cause. Comprehensive antimicrobial use databases should be set up to identify global hotspots where antimicrobials are disproportionately used. Furthermore, risk assessment approaches for preventing diseases, including developing and spreading of antimicrobial resistant bacteria, need to be established. In a similar manner, inexpensive and easy-to-use tests should be developed to detect antibiotic residues in foods rapidly. Finally, healthier approaches such as probiotics and herbal remedies should be used.

## Authors’ Contributions

MMJA, AKLD, and PIV: Conceptualized and designed review, literature search, and wrote the first manuscript draft. MMJA, SLA, SS, IK, DMS: Edited and revised the draft of the manuscript. All authors critically reviewed the manuscript and approved the final manuscript.
